# 567 Creatine Phosphokinase: A Novel Biomarker for Frostbite Severity

**DOI:** 10.1093/jbcr/iraf019.196

**Published:** 2025-04-01

**Authors:** Joshua Lemos, Cameron Gibson, Arek Wiktor, Scott Mueller

**Affiliations:** University of Colorado; University of Colorado; University of Colorado; Burn and Frostbite Center at UCHealth

## Abstract

**Introduction:**

Serum creatine phosphokinase (CPK) levels in injuries involving muscular damage, such as trauma, are used as an indicator of severity of injury. The role of CPK in frostbite injuries is unknown. We hypothesize that there is an association between serum CPK levels and the severity of frostbite injury.

**Methods:**

This is a single center retrospective chart review of patients admitted to an ABA verified burn center with an ICD-10 diagnosis of frostbite from October 2022 to July 2024. Patients were excluded from the study if they were under the age of 18, had end stage renal disease or dialysis requirements prior to admission, or no recorded CPK during evaluation. Peak CPK levels were compared to commonly known indicators of frostbite severity and interventions, such as Hennepin scores at presentation and on discharge, tissue plasminogen activator (tPA) administration, need for surgery and abnormal technetium-99 bone scan results. Data on the incidence of acute kidney injury (AKI) using the Kidney Disease Improving Global Outcomes guidelines was also collected. Statistical analysis was computed using Wilcoxon-rank sum and least squares linear regression for continuous variable comparison with statistical significance considered at p< 0.05.

**Results:**

A total of 95 patient charts were reviewed with 55 patients having recorded a serum CPK during their evaluation for frostbite. Median (interquartile range) of following variables: age 42 years (33.5, 58), initial Hennepin scores 20 (10,30), discharge Hennepin scores 5.5 (1.5, 17), initial CPK 556 U/L (181, 5542), peak CPK 630 (187, 6711), and serum creatinine 0.82 mg/dL (0.68, 1.21). Male sex, bone scan completion, and receipt of tPA were 84%, 84%, and 60%, respectively. Both initial (R=0.51, CI= [0.264, 0.684], p< 0.05) and discharge (R=0.31, CI= [0.037, 0.548], p< 0.05) Hennepin scores demonstrated a weak positive correlation with elevated peak serum CPK levels. Peak serum CPK levels were greater in those who received tPA (2369 [IQR 248-7200] vs. 317 [IQR 112-2063], p< 0.05), but not in those with abnormal perfusion by bone scan (p=0.5) or those that required surgery (2368 [187, 16429] vs 548 [185, 3804], p=0.21). Significantly higher CPK levels were present in those that developed AKI (3511 [IQR 509-14316] vs. 248 [IQR 141-3187], p< 0.05). There was no significant difference in age, unhoused status, alcohol or methamphetamine use between those who were treated with tPA or for those who developed AKI.

**Conclusions:**

Our results show that CPK levels are weakly correlated with Hennepin scores. Additionally, tPA administration and the development of AKI were associated with elevated CPK levels.

**Applicability of Research to Practice:**

Clinicians should consider checking CPK levels at the time of presentation as part of their overall work-up for frostbite. More research in the diagnostic efficacy of CPK values in frostbite patients is warranted for treatment algorithms.

**Funding for the Study:**

N/A

